# Therapeutic Hypothermia in Sudden Unexpected Postnatal Collapse: Feasibility, Risks, and Long-Term Outcomes—A Systematic Review

**DOI:** 10.3390/children12101422

**Published:** 2025-10-21

**Authors:** Enrico Cocchi, Aurora Brighi, Gina Ancora

**Affiliations:** 1 Department of Medical and Surgical Sciences (DIMEC), Alma Mater Studiorum—University of Bologna, 40126 Bologna, Italy; 2 Neonatal and Pediatric Intensive Care Unit, Department of Child and Maternal Health, AUSL Romagna, 48121 Ravenna, Italy; 3 Department of Medicine and Surgery, Alma Mater Studiorum—University of Bologna, 40126 Bologna, Italy; aurora.brighi2@studio.unibo.it; 4 Neonatal Intensive Care Unit, Department of Child and Maternal Health, AUSL Romagna, Infermi Hospital, 47923 Rimini, Italy; gina.ancora@auslromagna.it

**Keywords:** sudden unexpected postnatal collapse (SUPC), therapeutic hypothermia, skin-to-skin contact, neurodevelopmental outcome

## Abstract

**Highlights:**

**What are the main findings?**

- Therapeutic hypothermia has been increasingly applied to neonates with sudden unexpected postnatal collapse (SUPC), with survival and normal neurodevelopment reported in about half of cooled survivors.
- Most SUPC events occur during skin-to-skin contact or breastfeeding, often in primiparous mothers and without continuous supervision.

**What is the implication of the main finding?**

- While therapeutic hypothermia may offer benefit in selected SUPC cases, the evidence is limited to low-quality observational studies and remains inconclusive.
- Additional preventive strategies, including close monitoring during early skin-to-skin and breastfeeding, are essential to mitigate risk while maintaining the benefits of mother–infant contact.

**Abstract:**

**Background/Objectives**: Sudden unexpected postnatal collapse (SUPC) is a rare but catastrophic event affecting apparently healthy neonates during the first days of life. Therapeutic hypothermia has been increasingly applied in this setting due to pathophysiological overlap with hypoxic–ischemic encephalopathy, but its effectiveness remains uncertain. The aim of this review is to systematically identify, appraise, and synthesize the evidence on therapeutic hypothermia for SUPC. **Methods**: We searched MEDLINE, Scopus, Embase, Web of Science, and Cochrane up to February 2025. Eligible studies included term or near-term infants with SUPC within seven days of life who underwent therapeutic hypothermia. Data were extracted on demographics, collapse circumstances, therapeutic hypothermia protocol, mortality, seizures, neuroimaging, and neurodevelopment. **Results**: Thirteen studies were included, encompassing 70 infants. Most events occurred within two hours of life, during skin-to-skin or breastfeeding, and were strongly associated with primiparity. Therapeutic hypothermia was typically initiated within six hours of collapse, using whole-body cooling at 33–34 °C for 72 h. Mortality was approximately 10% (widely ranging from 0 to 50%). Seizures were frequent (70–90%), and MRI abnormalities were reported in about half of cases. Approximately half of survivors demonstrated normal neurodevelopment at one year. Study quality was low to moderate, and risk of bias substantial. **Conclusions**: Therapeutic hypothermia is feasible in SUPC and survival with favorable outcomes has been documented, but the certainty of evidence is very low. Given recurrent risk factors such as primiparity and early skin-to-skin/breastfeeding, enhanced vigilance and preventive strategies are essential. Therapeutic hypothermia should be considered case by case, ideally within specialized centers and supported by registries.

## 1. Introduction

Sudden unexpected postnatal collapse (SUPC) is a rare but catastrophic event that affects apparently healthy term or near-term neonates during the first seven days of life [1,2,3]. It is typically defined as a sudden cardiorespiratory arrest requiring resuscitation and intensive care admission in infants with a good initial adaptation after birth [3,4]. Reported incidence varies between 2 and 133 per 100,000 live births, although higher rates have been described in studies with active surveillance and centralized referral systems, suggesting that under-recognition may be common [1,4,5]. SUPC often occurs in the context of skin-to-skin contact, prone positioning, or breastfeeding, but the precise mechanisms remain unclear [3,6]. Mortality is high, and survivors are at considerable risk of neurological sequelae [7,8].

Therapeutic hypothermia has become the standard of care for neonates with hypoxic–ischemic encephalopathy following perinatal asphyxia [9,10]. Randomized controlled trials and meta-analyses have demonstrated its efficacy in reducing death and severe neurodevelopmental disability, and international guidelines recommend its initiation within six hours of birth [10,11,12]. The pathophysiological overlap between perinatal asphyxia and SUPC—both leading to acute hypoxic–ischemic brain injury—has led clinicians to extend the use of therapeutic hypothermia to infants with SUPC. Case reports and small series indicate that therapeutic hypothermia is feasible in this setting and may be associated with survival and favorable neurodevelopmental outcomes [8,13,14].

Although SUPC and hypoxic–ischemic encephalopathy share a final common pathway of hypoxic–ischemic brain injury, their underlying mechanisms and clinical contexts differ substantially. Hypoxic–ischemic encephalopathy typically results from intrapartum asphyxia with well-defined sentinel events and progressive encephalopathy soon after birth, whereas SUPC occurs in apparently healthy neonates who experience an unexpected collapse after a period of normal adaptation, often during skin-to-skin contact or breastfeeding. Consequently, SUPC may involve additional mechanisms such as airway obstruction, impaired arousal, or delayed recognition of hypoxia. These distinctions are important to delineate, as they may influence both the pathophysiological substrate and the potential response to therapeutic hypothermia.

However, the evidence supporting therapeutic hypothermia for SUPC is limited and extremely heterogeneous. The available literature consists almost exclusively of case reports and case series, with only a handful of retrospective cohorts and no randomized trials specific to this population [1,15]. While some cooled infants have demonstrated normal neurodevelopment at follow-up, others have died or suffered severe impairments, and direct comparisons with non-cooled infants are scarce [7,13,15,16]. Moreover, unlike classical hypoxic–ischemic encephalopathy, SUPC may be precipitated by alternative mechanisms such as intracranial hemorrhage, infection, or inborn errors of metabolism, in which therapeutic hypothermia could be less effective or even harmful [3,13].

Despite increasing use of therapeutic hypothermia in SUPC, no systematic synthesis of the evidence has yet clarified its effectiveness, safety, or impact on long-term neurodevelopmental outcomes. This systematic review aims to systematically identify, critically appraise, and synthesize the existing literature on the use of therapeutic hypothermia in neonates with SUPC, with the objective of informing clinical practice and highlighting areas for future research.

## 2. Materials and Methods

### 2.1. Protocol and Registration

This review was conducted according to the Preferred Reporting Items for Systematic Reviews and Meta-Analyses (PRISMA) 2020 statement [17]. The protocol was registered in the International Prospective Register of Systematic Reviews (PROSPERO; ID CRD420251154057 [18]).

### 2.2. Eligibility Criteria

We included studies of term or near-term neonates (>35 weeks’ gestation, reflecting the gestational range for which therapeutic hypothermia is currently recommended in perinatal hypoxic–ischemic encephalopathy) who experienced SUPC within the first 7 days of life and were treated with therapeutic hypothermia. SUPC was defined as a sudden collapse requiring resuscitation and intensive care admission in infants who had shown a good initial adaptation after birth.

- Study designs: case reports, case series, retrospective cohorts, prospective observational studies, and randomized controlled trials. We excluded conference abstracts, posters, and unpublished reports, as they did not provide sufficient methodological detail for critical appraisal.
- Population: neonates with SUPC as defined above.
- Intervention: any form of therapeutic hypothermia (whole-body or selective head cooling), regardless of start time, target temperature, or duration.
- Comparator: non-cooled SUPC infants, when reported.
- Outcomes: mortality before discharge, seizures, electroencephalographic findings, brain MRI results, and neurodevelopmental outcomes at ≥12 months.

It should also be acknowledged that neurological assessment and aEEG interpretation differ substantially in preterm infants, for whom background activity and maturational patterns are distinct from those observed at term. For this reason, the present review focused on infants >35 weeks’ gestation to ensure clinical and neurophysiological comparability with the population in which therapeutic hypothermia is currently validated.

Exclusion criteria: studies reporting collapse outside the first seven days of life, classic intrapartum hypoxic–ischemic encephalopathy without postnatal collapse, animal models, narrative reviews, and conference abstracts without sufficient primary data.

### 2.3. Information Sources and Search Strategy

We systematically searched MEDLINE, Scopus, Embase, Cochrane, and Web of Science from inception to February 2025. The search combined controlled vocabulary and free-text terms. The full search strategy is provided in Supplementary Materials. Reference lists of included studies and relevant reviews were screened manually to identify additional eligible articles. No language or date restrictions were applied.

### 2.4. Study Selection

Titles and abstracts were screened independently by two reviewers. Full texts of potentially eligible studies were then assessed against inclusion criteria. Discrepancies were resolved by discussion and, if necessary, by a third reviewer.

### 2.5. Data Extraction

Data were extracted independently by two reviewers using a piloted form. Extracted variables included the following:

- Study characteristics: first author, year, country, design, sample size.
- Patient demographics: gestational age, birth weight, sex, Apgar scores, age at collapse, circumstances (skin-to-skin, feeding, positioning).
- Clinical features: pH/base excess at admission, need for cardiopulmonary resuscitation, encephalopathy grade, seizures, aEEG findings.
- Therapeutic hypothermia: modality (whole-body/head), time from collapse to initiation, duration, target temperature, rewarming strategy, adverse events.
- Outcomes: survival to discharge, neuroimaging results, short-term neurological examination, and long-term neurodevelopmental outcomes.

Where studies reported both cooled and non-cooled infants, data were extracted separately. Table 1 summarizes study characteristics.

### 2.6. Risk of Bias Assessment

The methodological quality of included studies was assessed according to study design. Cohort-type studies were appraised using the Newcastle–Ottawa Scale, which evaluates selection, comparability, and outcome domains. Case reports and case series were evaluated with the Joanna Briggs Institute critical appraisal checklists. Randomized trials were screened through RoB 2. To facilitate comparability across designs, scores were subsequently harmonized into three categories: low, moderate, and high quality. For cohort studies assessed with the Newcastle–Ottawa Scale (maximum = 9 stars), this categorization used the following thresholds:

- Low quality: 0–4 stars;
- Moderate quality: 5–6 stars;
- High quality: 7–9 stars.

For case reports and case series evaluated using the Joanna Briggs Institute critical appraisal checklists:

- Low quality: 0–4/8 items fulfilled;
- Moderate quality: 5–6/8 items fulfilled;
- High quality: ≥7/8 items fulfilled.

These ranges were chosen to allow for consistent interpretation of methodological quality across study types.

### 2.7. Data Synthesis

Given the descriptive nature of most studies and the lack of comparable outcome metrics, quantitative synthesis or meta-analysis was not methodologically feasible. Studies were grouped by study design, time from collapse to initiation of therapeutic hypothermia, and availability of comparative non-cooled cases. Data were tabulated and summarized descriptively. A quantitative meta-analysis was considered but deemed not feasible because of the extreme heterogeneity across studies in terms of design (case report, case series, or cohort), sample size, inclusion criteria, timing and cause of collapse, therapeutic hypothermia protocols (type, duration, target temperature, and initiation time), and outcome definitions (mortality, seizures, MRI, neurodevelopment). Many reports lacked control groups or comparable denominators, preventing calculation of effect sizes or pooled estimates.

In addition to study-level quality assessment, the certainty of evidence across key outcomes was evaluated using the GRADE approach. Certainty was rated as high, moderate, low, or very low according to the five standard domains: risk of bias, inconsistency, indirectness, imprecision, and publication bias. The resulting summary is presented in Table 2.

## 3. Results

### 3.1. Study Selection

**Figure 1:**
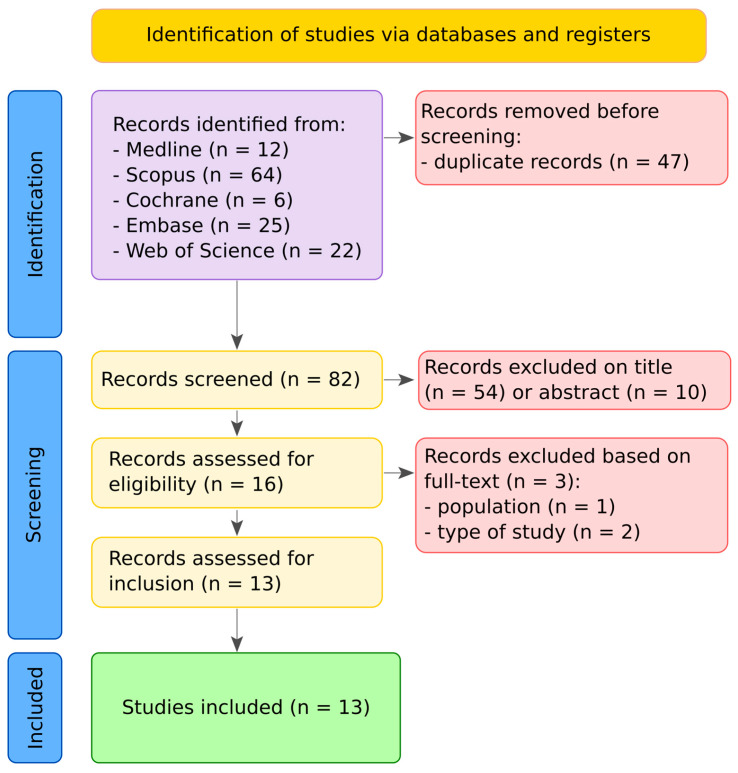
**PRISMA 2020 flow diagram summarizing the study selection process.** The search identified 129 records, of which 47 were duplicates. After title and abstract screening, 65 records were excluded. Full texts of 17 articles were assessed for eligibility, leading to the inclusion of 13 studies in the final review.

The search retrieved 129 records. After removing duplicates (n = 47), 82 titles and abstracts were screened. Following title screening, 54 records were excluded, and an additional 10 were excluded after abstract review. Full-text assessment was conducted for the remaining articles, resulting in 13 studies that fulfilled the eligibility criteria. The three records excluded in the last step were due to inclusion of patients that experienced life-threatening events later in life [16] and publication type [25,26]. The PRISMA 2020 flow diagram (Figure 1) depicts the study selection process. The agreement between the two independent reviewers was quantified using Cohen’s k coefficient = 0.86, indicating excellent concordance.

### 3.2. Study Characteristics

Thirteen studies were included, published between 2011 and 2023, across Europe, North America, Asia, and Australia [1,5,7,8,13,14,15,19,20,21,22,23,24]. Study designs comprised the following:

- Case reports (n = 4) [19,20,21,23];
- Case series (n = 4) [8,14,24];
- Cohort studies (n = 5) [1,5,7,13,15].

The total number of neonates with SUPC in those studies was N = 148; of those, N = 70 treated with therapeutic hypothermia. Table 1 summarizes study characteristics.

### 3.3. Patient Demographics and Event Circumstances

Most infants were born at term or near-term (range 36–42) with normal birth weight ranging from 2260 to 4580 g. The collapse typically occurred within the first two hours of life, though the range extended from 6 min to 72 h. Nearly all events took place during skin-to-skin or breastfeeding, frequently in the prone position, and often without continuous supervision. Primiparity was the most consistent risk factor, highlighted across almost all cases. Additional risk factors included maternal comorbidities (obesity, diabetes, thyroid disease, hypertension), sedation/analgesia during labor, maternal distraction (mobile phone use, fatigue, asleep), and unsafe infant positioning (prone, face against breast, co-bedding). Table 1 provides pooled demographic and event characteristics, while detailed characteristics are provided in Supplementary Materials.

### 3.4. Clinical Presentation and Initial Investigations

At admission, most infants required advanced cardiopulmonary resuscitation. Reported cord or post-resuscitation arterial pH values were often <7.0 with base excess below −12 mmol/L. aEEG was abnormal in the majority, showing burst suppression or low-voltage patterns.

### 3.5. Therapeutic Hypothermia Characteristics

Cooling was universally performed with whole-body hypothermia, 33–34 °C for 72 h, except for occasional selective head cooling [21] or shortened/milder protocols [8].

In all studies where details were provided, therapeutic hypothermia was delivered through servo-controlled cooling systems, ensuring precise temperature regulation in accordance with standard protocols for hypoxic–ischemic encephalopathy. Most studies applied hypoxic–ischemic encephalopathy eligibility criteria to SUPC [1,13,15]. The median time from collapse to therapeutic hypothermia initiation ranged from 5 min to 6 h [13,21,24], confirming feasibility of timely intervention in this scenario, even if in the majority of studies exact intervals were not provided.

### 3.6. Outcomes

#### 3.6.1. Survival and Short-Term Outcomes

Mortality rates varied widely, from 0% [7,13,14,19,20,21,22,23,24] to 50% [15]. Across all pooled cases, approximately 10% of cooled SUPC infants died, primarily those with severe acidosis and seizures.

#### 3.6.2. Seizures, aEEG and MRI

- Seizures: Very common—reported in 70–90% of cooled infants [7,8,13,15,20], though absent in a minority [14,21,23].
- aEEG: Almost all cooled infants showed moderate-to-severe abnormalities at admission. Importantly, early normalization (<36–48 h) strongly correlated with survival and favorable outcome [13,15].
- MRI: Abnormalities were present in about half of cases, mainly basal ganglia, thalami, or white matter lesions [1,8,15].

#### 3.6.3. Long-Term Neurodevelopment

Follow-up reporting was heterogeneous, but across studies that provided ≥12-month outcomes, roughly half to two-thirds of survivors had normal development, while a substantial minority had motor and/or cognitive impairment.

#### 3.6.4. Adverse Events

Across reports, no therapeutic hypothermia-related mortality was described. Reported complications were broadly consistent with standard therapeutic hypothermia experience in hypoxic–ischemic encephalopathy, but data were sparsely and inconsistently reported.

- Coagulopathy/bleeding risk: Mentioned as a concern, particularly where intracranial hemorrhage or bleeding diathesis was suspected—several teams withheld or stopped therapeutic hypothermia in this context (e.g., Filippi 2017 [1] excluded cooling in a case with pulmonary hemorrhage; Cornet 2014 [8] shortened therapeutic hypothermia in one rapidly improving infant and did not cool one with severe PPHN).
- Cardiac/skin issues: Occasional notes of arrhythmias and skin changes (pressure/cooling-related) were made, but without serious sequelae in the cooled SUPC cases described.
- Metabolic/etiologic cautions: Multiple authors emphasize caution or avoidance of therapeutic hypothermia when non-hypoxic mechanisms are identified or strongly suspected (e.g., inborn errors of metabolism, significant intracranial hemorrhage, severe pulmonary hypertension/injury)—see Smit 2014 [13] (non-standard cooled cohort noting complications when major ICH present), Filippi 2017 [1], and Cornet 2014 [8].
- Operational feasibility/safety: Single-center case series [14,20,21] and cohort work [7,13,24] report feasible application of standard 72 h whole-body therapeutic hypothermia (33–34 °C) without unexpected safety signals attributable to therapeutic hypothermia itself.

### 3.7. Risk of Bias Within Studies

The overall quality of the included evidence was limited by small sample sizes, heterogeneous designs, and frequent reliance on descriptive case reports or case series. Five studies with a cohort-like design [1,5,7,13,15] were appraised with the Newcastle–Ottawa Scale and generally achieved 7–8/9 stars, reflecting clear inclusion criteria, systematic follow-up, and standardized therapeutic hypothermia protocols. Their main limitations were the absence of comparators and limited control for potential confounders. The remaining studies [8,14,19,20,21,22,23,24] were case reports or small case series assessed with the Joanna Briggs Institute tools. These provided rich clinical detail but were moderate or low quality, largely due to anecdotal nature, risk of selection bias, and incomplete or short-term follow-up. Across all studies, reporting bias was common: adverse events, long-term neurodevelopmental outcomes, and precise timing of hypothermia initiation were inconsistently documented. Several case series lacked uniform neurological and neuroimaging assessments. In summary, risk of bias is substantial across the available evidence, reflecting both methodological and reporting limitations. The relative methodological strength of a few cohort-type studies does not offset the overall reliance on small case series and case reports, so the certainty of evidence remains low to very low.

## 4. Discussion

This systematic review identified 13 studies reporting the use of therapeutic hypothermia in neonates with SUPC. The evidence base consists almost exclusively of case reports and small case series, with few retrospective cohorts and no randomized controlled trials specific to this population. The overall evidence base is therefore extremely limited, heterogeneous, and insufficient to draw any firm conclusions on the efficacy of therapeutic hypothermia in SUPC. Across studies, therapeutic hypothermia was generally feasible and well tolerated. A portion of infants survived with normal neuroimaging and neurodevelopmental outcomes, while mortality remained substantial and long-term impairment was frequent. Comparative evidence between cooled and non-cooled infants was extremely scarce, precluding firm conclusions on efficacy. The certainty of the evidence was uniformly graded low to very low.

We applied the GRADE framework across the main outcomes (mortality, seizures, MRI findings, and neurodevelopment). Certainty of evidence was rated very low for all outcomes, primarily due to the observational design of included studies, small sample sizes, heterogeneous definitions, and incomplete follow-up. A concise visual summary of these ratings is provided in Table 2. This reinforces that current evidence should be interpreted as exploratory and hypothesis-generating rather than confirmatory.

Several studies consistently identified primiparity and the context of skin-to-skin contact and breastfeeding as recurrent circumstances in which SUPC occurred. In national surveillance and case-series data, the majority of mothers were primiparous, and collapses often arose during the first attempts at breastfeeding or while the infant was placed prone on the maternal chest, frequently without continuous supervision [5,7,8,15]. Although skin-to-skin care and early breastfeeding provide well-established benefits, these findings underscore that the early postnatal period carries a heightened risk of SUPC when protective vigilance may be lowest. Additional protective strategies are therefore warranted, particularly for primiparous mothers who may be less experienced in positioning and handling their newborn [27,28]. These include structured staff education, active observation during the first skin-to-skin and breastfeeding episodes, safe positioning with visible airways, and the avoidance of prone or obstructive postures. Implementation of such preventive measures has the potential to maintain the benefits of early mother–infant contact while mitigating the preventable risks associated with SUPC.

Interestingly, the overall mortality observed among cooled SUPC infants (~10%) appears lower than that typically reported in neonates with hypoxic–ischemic encephalopathy, where mortality rates approach 25–30% [11,12]. This discrepancy may reflect a selection bias toward milder cases of encephalopathy or preferential reporting of survivors in descriptive studies. Conversely, the proportion of infants presenting with seizures was markedly higher in SUPC (70–90% vs. 30–40%) [9,11]. This difference may be explained by systematic early aEEG or EEG monitoring following postnatal collapse and by pathophysiological distinctions between SUPC-related hypoxia and intrapartum asphyxia, including timing, duration, and possible reoxygenation dynamics. Overall, while both conditions share hypoxic–ischemic mechanisms, the clinical course and neurological expression of SUPC may differ substantially from typical perinatal hypoxic–ischemic encephalopathy.

Our findings align with previous narrative reviews and single-center experiences suggesting that therapeutic hypothermia is increasingly applied to SUPC, yet without robust evidence of benefit. Some series reported favorable outcomes in cooled infants, but others described deaths or severe disabilities despite therapeutic hypothermia. Limited comparative data from mixed cohorts hinted at better outcomes with cooling [7,8], though selection bias cannot be excluded. Centralization of care and increased awareness after the adoption of therapeutic hypothermia may also partly explain the apparent rise in reported cases, as suggested by surveillance data.

In the absence of high-quality evidence, decisions on therapeutic hypothermia for SUPC must be individualized. Early initiation of therapeutic hypothermia may be reasonable when SUPC leads to moderate or severe encephalopathy and hypoxic–ischemic injury is suspected, provided that alternative etiologies such as intracranial hemorrhage or metabolic disease have been excluded. Clinicians should be aware of the uncertain benefit-risk balance, the potential for complications, and the very low certainty of the supporting evidence. Careful parental counseling and transparent documentation of decision-making are essential.

### Limitations and Future Directions

Several limitations must be acknowledged. First, the evidence base is limited to observational designs, predominantly case reports and small series, inherently prone to selection and publication bias. Second, follow-up data were often incomplete, with only a minority of survivors assessed beyond 12 months. Third, heterogeneity in case definitions, timing and modality of cooling, and outcome reporting precluded meaningful meta-analysis. Moreover, long-term neurodevelopmental outcomes were rarely assessed with standardized tools (e.g., Bayley or Griffiths scales); thus, conclusions on developmental prognosis cannot be drawn. Future research should focus on establishing multicenter registries to systematically capture SUPC cases, with standardized definitions, detailed documentation of resuscitation and cooling protocols, and long-term neurodevelopmental follow-up. Comparative studies including cooled and non-cooled infants are urgently needed, although randomized trials may be challenging given the rarity and unpredictability of SUPC. Collaborative international networks will be essential to generate sufficient case numbers and improve the evidence base.

## 5. Conclusions

Therapeutic hypothermia is increasingly used in neonates with SUPC, and survival with favorable outcomes has been reported. However, the current evidence is limited to low-quality observational studies, and the effectiveness of therapeutic hypothermia in this context remains uncertain. Importantly, many events occurred in the setting of primiparity and during early skin-to-skin contact or breastfeeding, underscoring the need for enhanced vigilance and preventive strategies in these circumstances. Until more robust data are available, therapeutic hypothermia should be considered on a case-by-case basis after rapid exclusion of alternative causes, ideally within specialized centers, and accompanied by structured follow-up and registry enrollment.

## Figures and Tables

**Table 1:** **Characteristics and outcomes of studies reporting therapeutic hypothermia after sudden unexpected postnatal collapse (SUPC).** The table summarizes study design, sample size, therapeutic hypothermia (TH) initiation time, protocol, mortality, and neurodevelopmental outcomes at ≥12 months when available. Neurodevelopment is reported as the proportion of survivors with normal development; when follow-up was incomplete or absent this is indicated as NA. Study quality was harmonized across appraisal tools (Newcastle–Ottawa Scale for cohort studies; Joanna Briggs Institute checklists for case reports/series) into three categories: low, moderate, and high. Full details on gestational age, birth weight, circumstances of collapse, seizures, EEG/aEEG, and MRI findings are available in Supplementary Table S1.

Author (Year)	Country	Design	TH/SUPC (n)	Timing of TH (After Collapse)	TH Protocol	Mortality	Neurodevelopment ≥12 mo	Quality
Bedetti 2022 [14]	Italy	Case series	4/4	35–140 min	Whole-body	0%	3/4 normal at 24 mo	Moderate
Filippi 2017 [1]	Italy	Cohort	3/9	15–180 min	Whole-body	33%	NA	High
Cornet 2014 [8]	France	Case series	4/5	1–23 h	Whole-body or mild/shortened	25%	Normal in survivors (15–36 mo)	Moderate
Bei-Bei 2021 [19]	China	Case report	1/1	72 h	Whole-body	100%	Died	Low
Marín 2013 [20]	Spain	Case report	1/1	90 min	Whole-body	0%	Normal at 10 mo	Moderate
Smit 2015 [13]	UK	Case series	10/10	Minutes–32 h	Whole-body	0%	62% normal at 18–20 mo	High
Pejovic 2013 [7]	Sweden	Case series	4/26	15 min–20 h	Whole-body	0%	75% normal at 24 mo	High
Becher 2012 [5]	UK	Nat. cohort	3/45	6 min–10 h	Whole-body	33%	1 normal, 1 impaired, 1 died at 12 mo	High
Ancora 2013 [21]	Italy	Case report	1/1	75 min	Head cooling	0%	Normal at 12 mo	Moderate
Brito 2021 [15]	Spain	Retrospective cohort	22/22	20 min–23 h	Whole-body	50%	Limited, ND abnormal in many	High
Paul 2019 [22]	USA	Case series	2/5	60–150 min	Whole-body	0%	NA	Low
Mackay 2023 [23]	Australia	Case report	1/1	120 min	Whole-body	0%	Normal	Moderate
Echeverría 2019 [24]	Spain	Case series	14/18	<24 h	Whole-body	0%	Mixed, not uniform	High

**Table 2:** **GRADE assessment of the certainty of evidence for key outcomes following therapeutic hypothermia in neonates with sudden unexpected postnatal collapse**. Certainty of evidence was evaluated using the GRADE approach across five domains (risk of bias, inconsistency, indirectness, imprecision, and publication bias). Certainty was rated as high, moderate, low, or very low. All outcomes were derived from observational data without control groups; hence, the starting level was “low” and was further downgraded for serious limitations.

Outcome	N. of Studies (n. Cooled Infants)	Study Design	Overall Certainty	Reason	Summary of Evidence
Mortality	13 (n = 70)	Case reports, small cohorts	Very low	Serious risk of bias (observational, uncontrolled); high heterogeneity; imprecision due to small sample size; potential publication bias	Mortality ranged 0–50%, pooled ≈10%; cannot determine if TH reduces or alters mortality in SUPC
Seizures	8 (n = 47)	Case reports, small cohorts	Very low	Risk of bias; variable EEG detection; lack of comparator; inconsistency in reporting	Seizures frequent (70–90%); unclear association with outcome; may reflect early EEG monitoring
MRI abnormalities	3 (n = 29)	Small cohorts	Low to very low	Risk of bias; variability in imaging timing and interpretation	MRI abnormalities present in ~50% (BG, thalami, WM lesions); unclear prognostic role
Neurodevelopmental outcome	9 (n = 42)	Case reports, small cohorts	Very low	Risk of bias; incomplete or non-standardized follow-up; small samples; indirectness	About half to two-thirds had normal development; testing not standardized (Bayley, Griffiths rarely used)

## Data Availability

All data relevant to the study are publicly available and included within the article or provided as Supplementary Informations.

## Supplementary Materials

The following supporting information can be downloaded at: https://www.mdpi.com/article/10.3390/children12101422/s1, Table S1: Characteristics and outcomes of neonates with sudden unexpected postnatal collapse (SUPC) treated with therapeutic hypothermia.

## Abbreviations

The following abbreviations are used in this manuscript:

SUPC	Sudden Unexpected Perinatal Collapse
MRI	Magnetic Resonance Imaging
