# Supplementary material for: Therapeutic Hypothermia in Sudden Unexpected Postnatal Collapse: Feasibility, Risks, and Long-Term Outcomes—A Systematic Review
Source: Children (Basel). 2025 Oct 21;12(10):1422. doi: 10.3390/children12101422 (PMC12563370; doi:10.3390/children12101422)
Supplement: Supplementary file 1 [file children-12-01422-s001.zip › search-strategy.v2.pdf]

Search strategy. Search strategy for the systematic review. A comprehensive search was conducted in MEDLINE, Scopus, Embase, Web of Science, and Cochrane CENTRAL from inception to February 2025. Below details are reported.

Medline

1. Infant/ - 899,375

2. Infant, Newborn/ - 695,767

3. (Newborn or Neonat* or Infant).ab,ti,kw – 604,578

1 or 2 or 3 - 1,536,583

5. ("Sudden Unexpected Postnatal Collapse" or "SUPC" or "Neonatal Collapse" or "Unexpected Collapse" or "Postnatal Sudden" or "apparent life threatening event" or "ALTE" or "sudden unexpected death in infancy" or "SUDI" or "Brief Resolved Unexplained Event" or "BRUE" or "Acute Life Threatening Event" or "Brief Resolved Unexplained Symptoms" or "BRUS" or "Infant Acute Life Threatening Episode" or "Infant Critical Events").ab,ti,kw - 1,258

6. Hypothermia/ - 15,419

7. Induced Hypothermia/ - 22,489

8. Therapeutic Hypothermia/ - 22,489

9. Hypothermia, Therapeutic/ - 22,489

10. ("hypothermia" or "therapeutic hypothermia" or "cooling" or "freezing").ab,ti,kw - 128,178

11. 6 or 7 or 8 or 9 or 10 - 138,916

12. 4 and 5 and 11 – 12

13. 5 and 11 - 12

Scopus

TITLE-ABS-KEY ("Sudden Unexpected Postnatal Collapse" OR "SUPC" OR "Neonatal Collapse" OR "Unexpected Collapse" OR "Postnatal Sudden" OR "apparent life threatening event" OR

"ALTE" OR "sudden unexpected death in infancy" OR "SUDI" OR "Brief Resolved Unexplained Event" OR "BRUE" OR "Acute Life Threatening Event" OR "Brief Resolved Unexplained Symptoms" OR "BRUS" OR "Infant Acute Life Threatening Episode" OR "Infant Critical Events") AND TITLE-ABS-KEY ("hypothermia" OR "therapeutic hypothermia" OR "cooling" OR "freezing") - 64

Embase

1. newborn:ab,ti,kw OR neonat*:ab,ti,kw OR infant:ab,ti,kw – 797,030
2. 'sudden unexpected postnatal collapse':ab,ti,kw OR 'supc':ab,ti,kw OR 'neonatal collapse':ab,ti,kw OR 'unexpected collapse':ab,ti,kw OR 'postnatal sudden':ab,ti,kw OR 'apparent life threatening event':ab,ti,kw OR 'alte':ab,ti,kw OR 'sudden unexpected death in infancy':ab,ti,kw OR 'sudi':ab,ti,kw OR 'brief resolved unexplained event':ab,ti,kw OR 'brue':ab,ti,kw OR 'acute life threatening event':ab,ti,kw OR 'brief resolved unexplained symptoms':ab,ti,kw OR 'brus':ab,ti,kw OR 'infant acute life threatening episode':ab,ti,kw OR 'infant critical events':ab,ti,kw – 1,674
3. 'hypothermia':ab,ti,kw OR 'therapeutic hypothermia':ab,ti,kw OR 'cooling':ab,ti,kw OR 'freezing':ab,ti,kw - 154,178
4. #1 AND #2 AND #3 - 25

Web of Science

1: TS=(Newborn or Neonat* or Infant) - Results: 804,718

2: TS=(“Sudden Unexpected Postnatal Collapse” or “SUPC” or “Neonatal Collapse” or “Unexpected Collapse” or “Postnatal Sudden” or “apparent life threatening event” or “ALTE” or “sudden unexpected death in infancy” or “SUDI” or “Brief Resolved Unexplained Event” or “BRUE” or “Acute Life Threatening Event” or “Brief Resolved Unexplained Symptoms” or “BRUS” or “Infant Acute Life Threatening Episode” or “Infant Critical Events”) - Results: 2039

3: TS=("hypothermia" or "therapeutic hypothermia" or "cooling" or "freezing") – 515,289

4: #2 AND #3 - Results: 22

Cochrane library

#1 “Sudden Unexpected Postnatal Collapse” or “SUPC” or “Neonatal Collapse” or “Unexpected Collapse” or “Postnatal Sudden” or “apparent life threatening event” or “ALTE” or “sudden unexpected death in infancy” or “SUDI” or “Brief Resolved Unexplained Event” or “BRUE” or “Acute Life Threatening Event” or “Brief Resolved Unexplained Symptoms” or “BRUS” or “Infant Acute Life Threatening Episode” or “Infant Critical Events” - 212

#2 MeSH descriptor: [Hypothermia] explode all trees - 977

#3 MeSH descriptor: [Hypothermia, Induced] explode all trees – 1,333

#4 "hypothermia" or "therapeutic hypothermia" or "cooling" or "freezing" – 9,576

#5 #2 or #3 or #4 – 9,576

#6 #1 and #5 - 6
