# Supplementary material for: Therapeutic Hypothermia in Sudden Unexpected Postnatal Collapse: Feasibility, Risks, and Long-Term Outcomes—A Systematic Review
Source: Children (Basel). 2025 Oct 21;12(10):1422. doi: 10.3390/children12101422 (PMC12563370; doi:10.3390/children12101422)
Supplement: Supplementary file 1 [file children-12-01422-s001.zip › supplementary-table.pdf]

Supplementary Table S1. Characteristics and outcomes of neonates with sudden unexpected postnatal collapse (SUPC) treated with therapeutic hypothermia. Reported variables include gestational age (GA), birth weight, timing and circumstances of SUPC, maternal or perinatal risk factors, therapeutic hypothermia (TH) protocols, and key outcomes (mortality, seizures, EEG, MRI, and neurodevelopment). Quality appraisal was performed with the Newcastle-Ottawa Scale (for cohort-type studies) or the Joanna Briggs Institute checklists (for case reports and series), and harmonized as low, moderate, or high.

Author	Year	Country	Yype	SUPC (n.)	TH (n.)	GA	Weight	Timing SUPC	Circumstance	Risk Factor	TH protocol	Mortality	Seizure	EEG	MRI	Neurodevelopment	Quality
Bedetti et al.	2022	Italy	Case series	4	4	37-42	2740-3500	35-140 min	skin-to-skin / prone	Primiparity common	Standard whole-body	0	0	Abnormal	Ranged from normal (cases 2–3) to mild WM lesion (case 4) to severe BG/thalamic injury (case 1)	At 24 months: normal (75%)	Moderate
Filippi et al.	2017	Italy	Cohort	9	3	37-40	2920-4259	15-180 min	skin-to-skin / breastfeedin	Primiparity common	Standard whole-body	33%	66%	Abnormal	Normal to severe	NA	High

									g								
Cornet et al.	2014	France	Case series	5	4	38-41	2920-3270	1-23 hours	skin-to-skin / breastfeeding	Primiparity in 60 %	2 standard whole-body, 1 mild (34.5 °C), 1 shortened (15 h)	25 %	80 %	Abnormal	Normal (50 %), mild atrophy (25 %)	Normal at 15–36 months	Moderate
Bei-Bei et al.	2021	China	Case report	1	1	38	3400	72 hours	prone	Primiparity	Standard whole-body	0	100 %	Burst-suppression	Normal	Died	Low
Marin et al.	2013	Spain	Case report	1	1	40	3180	90 min	breastfeeding	Primiparity	Standard whole-body	0	100 %	Burst-suppression	Basal ganglia and thalamic lesions at day 11, absent on repeat MRI at 9 months.	Normal at 10 months	Moderate

Smit et al.	2014	UK	Case series	10	10	37-42	2460-4430	minutes up to 32 h	mostly breastfeeding	Primiparity common	Standard whole-body	0	96%	Abnormal	Abnormalities reported in some	Normal at ~18–20 months (62%)	High
Pejovic et al.	2013	Sweden	Case series	26	4	36-42	2575-4580	15 min – 20 h after birth	Mostly prone, skin-to-skin or breastfeeding	Primiparity (majority)	Standard whole-body	0	100%	Abnormal	Abnormalities reported in some	Normal at 24 months (75%)	High
Becher et al.	2011	UK	Prospective national study	45	3	37-42	2260-4030	6 min to 10 hours	Often breastfeeding, prone, skin-to-skin	Primiparity (77% of mothers)	Standard whole-body	33%	100%	Abnormal	Abnormalities reported in some	At 12 months, normal (33%), neurodisability (33%), died (33%)	High
Ancora	2013	Italy	Case report	1	1	37	2765	75 min	skin-to-skin	Primiparity	Selective head cooling	0	0	Burst-suppression	NA	Normal at 12 months	Moderate
Brito et al.	2021	Spain	Retrospective	22	22	38-40	2335-4250	20 min to 23	Mostly skin-to-	Primiparity	Standard who	50%	82%	Abnormal (95	Abnormal in 54	NA	High

			multicenter					hours	skin/breastfeeding	(86%)	le-body			%)	%		
Paul et al.	2019	USA	Case series	5	2	37-38	2670-3030	60-150 min	skin-to-skin/ breastfeeding	Primiparity	Standard whole-body	0	NA	NA	NA	NA	Low
Mackay et al.	2023	Australia	Case report	1	1	36	NA	120 min	skin-to-skin	Primiparity	Standard whole-body	0	0	Normal	Normal	Normal	Moderate
Echevería et al.	2019	Spain	Case series	18	14	>36	normal	<24 hours	skin-to-skin/ prone	Primiparity frequent	Standard whole-body	0	78	Abnormal	Mild white matter injury to severe basal ganglia/thalamic injury	Follow-up not uniform	High
